# *At*FUT4 and *At*FUT6 Are Arabinofuranose-Specific Fucosyltransferases

**DOI:** 10.3389/fpls.2021.589518

**Published:** 2021-02-09

**Authors:** Maria J. Soto, Pradeep Kumar Prabhakar, Hsin-Tzu Wang, Jason Backe, Digantkumar Chapla, Max Bartetzko, Ian M. Black, Parastoo Azadi, Maria J. Peña, Fabian Pfrengle, Kelley W. Moremen, Breeanna R. Urbanowicz, Michael G. Hahn

**Affiliations:** ^1^Lawrence Berkeley National Laboratory, DOE Joint Genome Institute, Berkeley, CA, United States; ^2^The Complex Carbohydrate Research Center, University of Georgia, Athens, GA, United States; ^3^Department of Plant Biology, University of Georgia, Athens, GA, United States; ^4^Center for Bioenergy Innovation, Oak Ridge National Laboratory, Oak Ridge, TN, United States; ^5^Department of Biochemistry and Molecular Biology, University of Georgia, Athens, GA, United States; ^6^Department of Biomolecular Systems, Max-Planck-Institute of Colloids and Interfaces, Potsdam, Germany; ^7^Department of Chemistry, University of Natural Resources and Life Sciences, Vienna, Austria

**Keywords:** Fucosyltransferase, arabinogalactan protein, *At*FUT1, *At*FUT4, *At*FUT6, GT37, plant cell wall, hydroxyproline-rich glycoprotein

## Abstract

The bulk of plant biomass is comprised of plant cell walls, which are complex polymeric networks, composed of diverse polysaccharides, proteins, polyphenolics, and hydroxyproline-rich glycoproteins (HRGPs). Glycosyltransferases (GTs) work together to synthesize the saccharide components of the plant cell wall. The *Arabidopsis thaliana* fucosyltransferases (FUTs), *At*FUT4, and *At*FUT6, are members of the plant-specific GT family 37 (GT37). *At*FUT4 and *At*FUT6 transfer fucose (Fuc) onto arabinose (Ara) residues of arabinogalactan (AG) proteins (AGPs) and have been postulated to be non-redundant AGP-specific FUTs. *At*FUT4 and *At*FUT6 were recombinantly expressed in mammalian HEK293 cells and purified for biochemical analysis. We report an updated understanding on the specificities of *At*FUT4 and *At*FUT6 that are involved in the synthesis of wall localized AGPs. Our findings suggest that they are selective enzymes that can utilize various arabinogalactan (AG)-like and non-AG-like oligosaccharide acceptors, and only require a free, terminal arabinofuranose. We also report with GUS promoter-reporter gene studies that *AtFUT4* and *AtFUT6* gene expression is sub-localized in different parts of developing *A. thaliana* roots.

## Introduction

The plant cell wall is a complex polymeric network composed of diverse polysaccharides, proteins, polyphenolics, and hydroxyproline-rich glycoproteins (HRGPs). The polysaccharide and glycoprotein components of the cell wall confer a range of important functions, from structural integrity to cell-cell communication (Darvill et al., 1985). These complex glycopolymers are comprised of numerous monosaccharide building blocks, such as glucose (Glc), galactose (Gal), arabinose (Ara), galacturonic acid (GalA), xylose (Xyl), rhamnose (Rha), and fucose (Fuc), among others. The diversity of plant cell wall glycans can be attributed to the various linkage combinations, conformations, and degrees of polymerization in which these monosaccharides can be organized to form polymers.

Fucose is a deoxyhexose sugar that is commonly found on the side-chains and core regions of glycans in plants, bacteria, fungi, vertebrates, and invertebrates. In the cell walls of plants, Fuc is a component of the pectic polysaccharides, rhamnogalacturonan I and rhamnogalacturonan II (RG-I and RG-II; Atmodjo et al., 2013), the hemicellulose xyloglucan (XyG; Pauly and Keegstra, 2016), and arabinogalactan proteins (AGPs; Tan et al., 2012). RG-I consists of a backbone of repeating disaccharide units of [*α*-(1,4)-d-GalA-α-(1,2)-l-Rha]*_n_* with sidechains composed of variously linked Ara and Gal residues, with Fuc and glucuronic acid (GlcA) present to a lesser extent (Ridley et al., 2001; Willats et al., 2001; Mohnen, 2008). RG-II is the most structurally complex of the pectins and all known cell wall structures, and consists of a homogalacturonan (HG) backbone of α-(1,4)-linked GalA that is further substituted by side branches (denoted A–F) consisting of 12 different monosaccharides, including Fuc (Ndeh et al., 2017). XyG is a hemicellulosic polysaccharide composed of a β-(1,4)-linked Glc backbone with side-chains initiated by α-(1,6) linked Xyl residues; these are often further decorated with Gal and Fuc residues (Pauly and Keegstra, 2016). Together, primary cell wall polysaccharides, including pectins and hemicelluloses, are deeply implicated to be involved in plant growth, cell expansion, wall porosity, and several other functions (Ridley et al., 2001; Willats et al., 2001; Mohnen, 2008; Pauly and Keegstra, 2016).

Arabinogalactan proteins are extracellular glycoproteins of the HRGP superfamily, and have been postulated to have roles in diverse plant growth and developmental responses, including cell expansion and division, hormone signaling, and abiotic stress responses (Seifert and Roberts, 2007; Tan et al., 2012). AGPs are extensively *O*-glycosylated and consist of a core protein backbone rich in proline (Pro), alanine (Ala), serine (Ser), and threonine (Thr), and carbohydrate moieties that account for 90–98% of the total weight (Tan et al., 2012). To allow for *O*-glycosylation, Pro residues of the protein backbone are post-translationally modified by prolyl hydroxylation, converting Pro to hydroxyproline (Hyp; Seifert and Roberts, 2007). AGPs are then *O*-glycosylated on non-contiguous Hyp residues with arabinogalactan (AG) oligosaccharides composed mainly of a β-(1,3) linked Gal backbone substituted with β-(1,6)-linked Gal side-chains that are further modified with α-(1,3) or α-(1,5)-linked Ara residues. Additional side chain modifications, including Fuc, Rha, and 4-*O*-methylated GlcA have also been identified in some species and plant tissues (Seifert and Roberts, 2007; Knoch et al., 2014; Smith et al., 2020).

**Figure 1:**
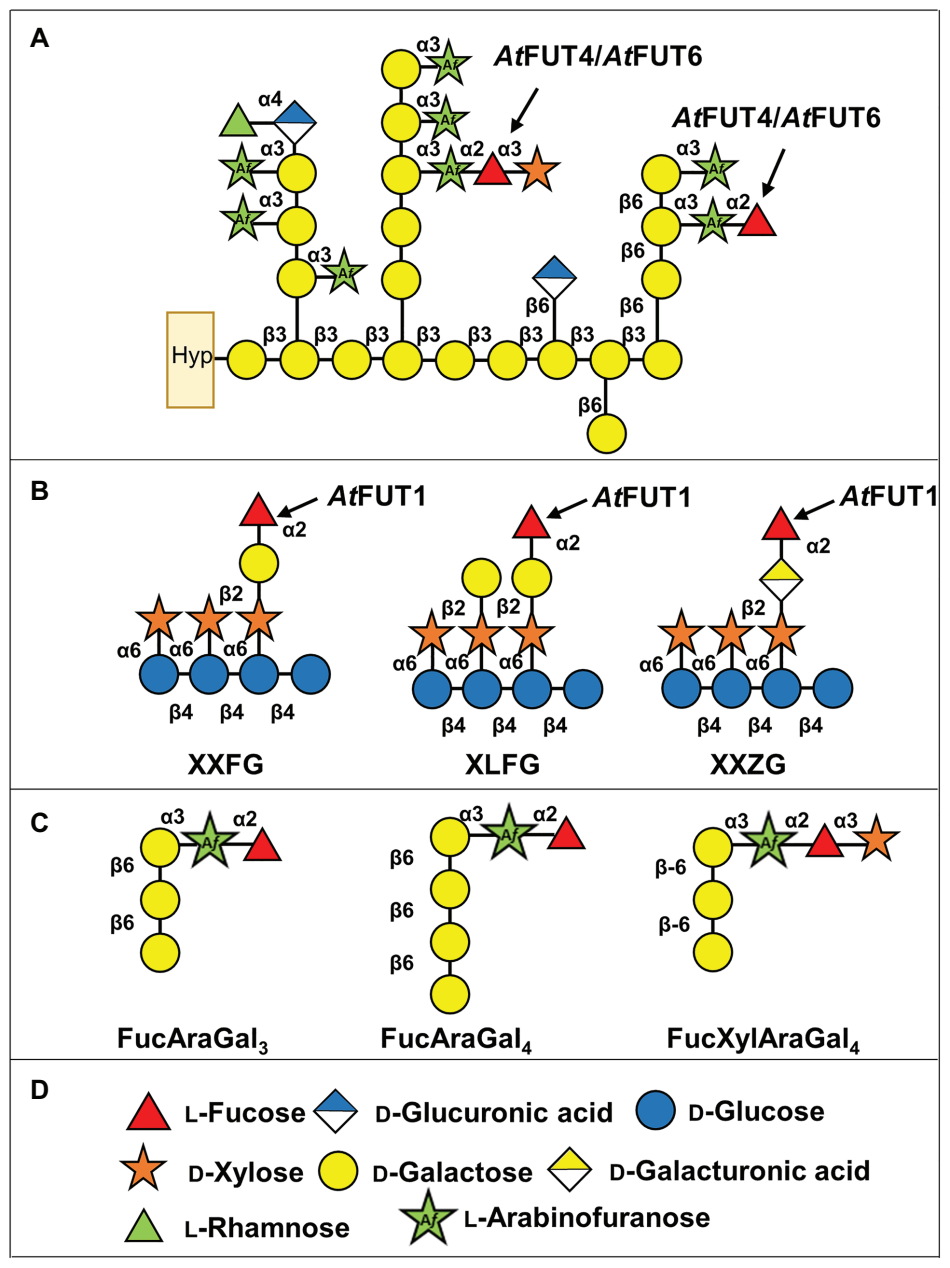
Scheme showing the structures of xyloglucan (XyG) and arabinogalactan (AG) proteins (AGPs) from *Arabidopsis thaliana* with the predicted activities of *At*FUT1 and *At*FUT4/*At*FUT6. **(A)** Representation of *A. thaliana* AGPs, and the putative activities of *At*FUT4 and *At*FUT6. Hyp, hydroxyproline. **(B)** Fucosylated XyG oligosaccharides derived from *A. thaliana* XyG (Peña et al., 2012; Tuomivaara et al., 2015). **(C)** Fucosylated AGP side chains derived from *A. thaliana* AGPs (Tryfona et al., 2012, 2014). **(D)** Symbol legend.

The addition of Fuc onto plant polysaccharides and proteoglycans is carried out by fucosyltransferases (FUTs). FUTs are glycosyltransferases (GTs) that catalyze the transfer of Fuc from guanidine 5'-diphosphate-β-l-fucose (GDP-Fuc) onto a suitable acceptor substrate, typically a glycan or protein. Although largely understudied in plants, known and putative FUTs are highly prevalent in many plant genomes (Soto et al., 2019). Interestingly, unlike the FUTs found in vertebrates and invertebrates that form clades based on predicted function (Martinez-Duncker et al., 2003), the FUTs in plants form terminal clades largely composed of single or closely related species (Soto et al., 2019). The model plant species *Arabidopsis thaliana* has 13 FUTs, 10 of which are classified as members of GT family 37 (GT37) according to the Carbohydrate-Active enZYmes (CAZy) database, and they are all predicted to be Golgi-localized type-II transmembrane proteins (Sarria et al., 2001; Lombard et al., 2014). Thus far, four out of the 10 GT37 FUTs from *A. thaliana* have been functionally characterized: *At*FUT1, *At*FUT4, *At*FUT6, and *At*FUT7 (Figures 1A,B; FUT7; Ruprecht et al., 2020).

*At*FUT1 is the most well-characterized member of the GT37 family. It catalyzes the regiospecific transfer of a Fuc residue to *O*-2 of the Gal of the L-side chain closest to the reducing end of a XyG subunit, to form the triglycosyl side-chain α-l-Fuc*p*-(1,2)-β-d-Gal*p*-(1,2)-α-d-Xyl*p*, also known as the F side-chain according to the accepted XyG nomenclature (Fry et al., 1993; Tuomivaara et al., 2015; Figure 1B). Structural characterization of *At*FUT1 by X-ray crystallography (Rocha et al., 2016; Urbanowicz et al., 2017), combined with investigations using molecular dynamic and quantum computations, suggests that *At*FUT1 fucosylates XyG using a water-mediated catalytic mechanism (Urbanowicz et al., 2017). The unusual phylogenetic relationship that plant FUTs exhibit has made identifying functionally homologous FUTs in other species difficult. For example, the functional homolog to *At*FUT1 in rice, *Os*MUR2, is phylogenetically distinct from *At*FUT1 and was identified through co-expression analyses based on the homologous genes in *A. thaliana* that had been shown to be involved in XyG biosynthesis (Liu et al., 2015). *At*FUT4 and *At*FUT6 have been less extensively studied, and were first identified based on their sequence similarity to *At*FUT1 (Sarria et al., 2001). Initial studies on *At*FUT4 and *At*FUT6 relied on the expression of these enzymes by transient transfection in Bright Yellow-2 (BY-2) suspension-cultured tobacco cells. Digitonin-solubilized extracts from microsomal membranes of transgenic tobacco BY2 cells overexpressing either *At*FUT4 or *At*FUT6 were able to incorporate [^14^C]Fuc from GDP-[^14^C]Fuc onto AGP acceptors, suggesting that these enzymes were AGP-specific FUTs (Wu et al., 2010). In this initial study, the *At*FUT4-enriched extract was able to fucosylate AGP fractions obtained from BY-2 tobacco suspension cell lines expressing the *At*FUT6 protein. The same inverse relationship was also reported for *At*FUT6, whereby the *At*FUT6-enriched extract from BY-2 tobacco suspension cells was able to fucosylate AGP fractions from lines expressing the *At*FUT4 protein. These apparent differences in activity led to the conclusion that *At*FUT4 and *At*FUT6 are both AGP-specific, but non-redundant, FUTs that potentially transfer Fuc onto different sites of AGP acceptors (Wu et al., 2010). This notion was further supported by the differing expression patterns exhibited for the corresponding genes, with *AtFUT4* being expressed in both roots and leaves, while *AtFUT6* is solely expressed in roots (Sarria et al., 2001; Liang et al., 2013; Tryfona et al., 2014). Interestingly, similar expression patterns have been shown for the *A. thaliana* arabinogalactan methyltransferases (AGMs) *AtAGM1* and *AtAGM2*, which are required for 4-*O*–methylation of terminal GlcA of AGPs (Smith et al., 2020; Temple et al., 2019).

Enzymatic derivatization and subsequent structural characterization of root and leaf AGPs from wild-type (WT) *A. thaliana*, as well as from *fut4*, *fut6*, and *fut4/fut6* single and double mutant plants, led to the identification of three types of fucosylated oligosaccharides from both tissues: FucAraGal_3_, FucAraGal_4_, and FucXylAraGal_3_ (Tryfona et al., 2012, 2014; Figure 1C). Detailed analyses of the AGPs produced by the *fut4*, *fut6*, and *fut4/fut6* mutants suggested that these gene products are non-redundantly involved in the transfer of terminal (1,2)-fucosyl residues to (1,3)-linked α-l-Ara*f* substituents of β-(1,6)-linked galactan side chains of AG, forming an α-l-Fuc*p*-(1,2)-α-l-Ara*f*-(1,3)-β-Gal*p*-(1,6)-β-Gal*p*-(1,6)-Gal*p* sidechain (FucAraGal_3_), which can be further modified by the addition of Xyl (Tryfona et al., 2012, 2014; Figure 1C). Further structural analyses of the AGPs of these mutants demonstrated that *AtFUT4* is solely responsible for the production of these fucosylated oligosaccharides in leaves, while both *AtFUT4* and *AtFUT6* are required in roots (Tryfona et al., 2012, 2014; Liang et al., 2013). The inconsistencies between these two previous studies led to the updated conclusion that *At*FUT4 and *At*FUT6 are both AGP-specific, but partially redundant. Recently, we performed a screen to evaluate acceptor substrate specificity using a glycan array-based assay, which showed that *At*FUT7 shares similar acceptor substrate specificity with *At*FUT4 and *At*FUT6, and all three enzymes fucosylate arabinofuranose residues α-(1,3)-linked to galactose (Ruprecht et al., 2020). In this report, we have done additional biochemical characterization of *At*FUT4 and *At*FUT6, combined with detailed structural analyses of their fucosylated reaction products, demonstrating that *At*FUT4 and *At*FUT6 are selective enzymes with regard to their acceptor substrate specificity and are fully-redundant in their recognition of various AG-like and non-AG-like oligosaccharide acceptor substrates. We also show that *AtFUT6* is expressed primarily in the root cap and meristematic zones of the root, while *AtFUT4* is present in the maturation and elongation zones of the root. The sub-localization of both enzymes to different regions of the roots may explain the requirement of both *At*FUT4 and *At*FUT6 for proper root AGP fucosylation previously reported.

## Materials and Methods

### Production of Constructs for Heterologous Expression in Human Embryonic Kidney 293 Cells

Full length cDNA clones obtained from The Arabidopsis Information Resource (TAIR) were used as templates to amplify truncated coding region sequences, excluding the predicted transmembrane domain, of *At*FUT4 and *At*FUT6. *At*FUT4 was truncated at amino acid residue 54, while *At*FUT6 was truncated at amino acid residue 42. To generate Gateway entry clones, *attB*-PCR products were created using two-step adapter PCR (Prabhakar et al., 2020). Primer sequences can be found in Supplementary Table S1.

Following the first round of PCR amplification, the universal primers attB_AdapterF, 5'GGGGACAAGTTTGTACAAAAAAGCAGGCTCTGA AAACTTGTA CTTTCAAGGC-3', and attB_Adapter-R, 5'-GGGGACCA CTTTGTACAAGAAAG CTGGGTC-3', were used to complete the *attB* recombination sites and introduce a tobacco etch virus (TEV) protease cleavage site for subsequent protein purification steps. The *attB*-PCR products were then cloned into a plasmid cloning vector, pDONR221, using the Gateway BP Clonase II Enzyme Mix (ThermoFisher Scientific), according to the manufacturer’s instructions. Expression clones were then created by recombining the entry clones into the pGEn2-DEST destination vector (Moremen et al., 2018) using the Gateway LR Clonase II Enzyme Mix (ThermoFisher Scientific), according to the manufacturer’s instructions. Fusion proteins produced using the pGEn2-DEST vector yield a fusion protein consisting of an *N*-terminal NH_2_-signal sequence, 8xHis tag, AviTag recognition site, superfolder GFP (sfGFP), and the seven amino acids comprising the TEV protease recognition site, followed by the truncated coding regions of *At*FUT4 or *At*FUT6.

### Protein Expression and Purification

The expression of the GFP-*At*FUT4 and GFP-*At*FUT6 recombinant enzymes was carried out by transiently transfecting HEK293 cells (Freestyle 293-F cells, ThermoFisher Scientific), as previously described (Urbanowicz et al., 2017; Moremen et al., 2018; Prabhakar et al., 2020). Chromatography experiments were performed on an AKTA FPLC System (GE Healthcare, https://www.gehealthcare.com). Prior to loading the Nickel-column, the media were adjusted to contain HEPES (25 mM, pH 7.2), sodium chloride (400 mM), and imidazole (20 mM). Small-scale purification of secreted 8xHis-GFP recombinant enzymes from HEK293 cells was carried out with HisTrap HP columns (GE Healthcare) following the manufacturer’s instructions. Protein cross-contamination was avoided by purifying each enzyme, GFP-*At*FUT4 or GFP-*At*FUT6, on individual 1-ml HisTrap columns that were washed before use to remove weakly bound Ni^2+^ ions. Protein purification procedures were performed as previously described (Urbanowicz et al., 2017; Prabhakar et al., 2020). GFP-*At*FUT1 was similarly prepared (Urbanowicz et al., 2017), and was included in this study as a positive control.

### Oligosaccharides Tested as Possible Acceptor Substrates

**Figure 2:**
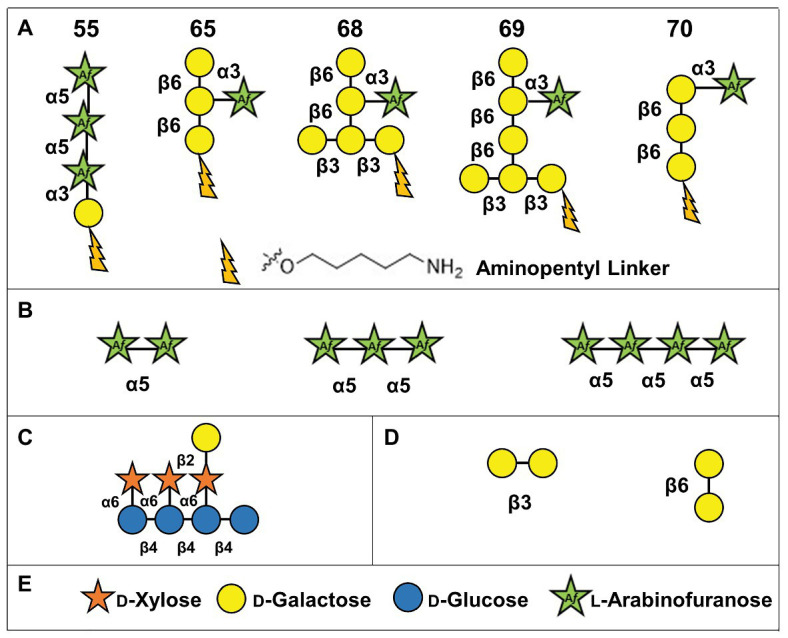
Structures of oligosaccharide acceptor substrates used in this study. **(A)** Synthetic arabinogalactan oligosaccharides numbered according to Ruprecht et al. (2020). **(B)** α-(1,5)-linked arabinan oligosaccharides. **(C)** Xyloglucan XXLG oligosaccharide. **(D)** β-(1,3)-linked galactobiose and β-(1,6)-linked galactobiose. **(E)** Symbol legend.

In order to assay the activities of GFP-*At*FUT1, GFP-*At*FUT4, and GFP-*At*FUT6, a series of chemically synthesized and commercially available oligosaccharides were tested as potential acceptor substrates. Five oligosaccharides were chemically synthesized by automated glycan assembly in the laboratory of Dr. Fabian Pfrengle (Bartetzko et al., 2015; Bartetzko and Pfrengle, 2019), and are indicated in this paper by numbers as 55, 65, 68, 69, and 70 according to the nomenclature utilized in Ruprecht et al. (2020). These oligosaccharides were selected due to their structural similarities to the previously identified fucosylated sidechains of WT *A. thaliana* AGPs: FucAraGal_3_, FucAraGal_4_, and FucXylAraGal_3_ (Tryfona et al., 2012, 2014; Figures 1C, 2A). A XyG oligosaccharide mixture consisting of XXXG, XXLG, and XLLG, named according to the standardized XyG nomenclature, were prepared as described (Tuomivaara et al., 2015; Figure 2C) from XyG isolated from the *A. thaliana mur1* mutant, which lacks fucosylated polysaccharides. Finally, three commercially available α-(1,5)-linked arabinan oligosaccharides (Figure 2B; arabinobiose, arabinotriose, and arabinotetraose) and two galactan oligosaccharides [Figure 2D; β-(1,3)-linked galactobiose and β-(1,6)-linked galactobiose] were obtained from Megazyme (Ireland).

### Enrichment of AGPs From *Arabidopsis thaliana* Plants

At least two mutant alleles of *At*FUT4 and *At*FUT6 were previously characterized by our group and collaborators (Liang et al., 2013). For the purpose of the experiments described here for substrate isolation, one mutant line (*fut4*, SAIL_284_B05) for *AtFUT4* (At2g15390) and one mutant line (*fut6*, SALK_099500) for *AtFUT6* (At1g14080) and a double mutant generated from these lines were selected based on our prior genetic, phenotypic, and chemotypic analyses (Liang et al., 2013; Tryfona et al., 2014). Root and aerial plant tissues for isolation of AGPs were generated as recently described (Smith et al., 2020) from a minimum of 30 pooled plants. Alcohol-insoluble residues (AIR) of wildtype (WT, Col-*0*) and *fut4*, *fut6*, and *fut4/fut6 A. thaliana* plants were prepared using a standard protocol (Pattathil et al., 2012). AGPs were extracted from the cell walls by resuspending AIR at 10 mg/ml in 50 mM sodium acetate, pH 5, and mixed for 16 h on a rotary shaker (200 rpm) at 55°C according to the “hot buffer” method described by Smith et al. (2020). The suspension was centrifuged at 3,000 × *g* and the supernatant was dialyzed against 16 L of deionized water four times for 24 h at room temperature. The dialysates were lyophilized and used as acceptor substrates in GDP-Glo™ GT Assays at a final concentration of 0.5 mg/ml (see below).

### Matrix-Assisted Laser Desorption/Ionization-Time of Flight Mass Spectrometry

The saccharide products of FUT reactions were analyzed by matrix-assisted laser desorption/ionization-time of flight mass spectrometry (MALDI-TOF MS) on a Microflex LT spectrometer (Bruker). Enzyme reactions were incubated at 24°C in 25 mM HEPES, pH 7.2, and consisted of 100 ng of enzyme, 100 μM of GDP-Fuc (Promega), and 250 μM of acceptor substrate for the AG oligosaccharides (55, 65, 68, 69, and 70; Figure 2A). For the α-(1,5)-linked arabinan oligosaccharides (Figure 2B) and the β-(1,3)-linked and β-(1,6)-linked galactobiose oligosaccharides (Figure 2D; Megazyme, https://www.megazyme.com/), 1 mM of GDP-Fuc and 2 mM of each acceptor were used. Control assays contained the same components except for the enzyme. After overnight incubation, 5 μl aliquots of the reactions were incubated with 1 μl of Dowex-50 cation exchange resin (Bio-rad) for 1 h, followed by centrifugation. One microliter of the supernatants were then mixed with 1 μl of matrix solution [20 mg/ml of 2,5-dihydroxbenzoic acid (DHB) in 50% (v/v) methanol] and spotted and crystallized on the target plate. A minimum of 200 laser shots were summated in order to generate the positive-ion spectra that were recorded. Due to the small masses of the oligosaccharides being analyzed, no clean-up steps such as de-salting, which minimize background signals from the buffer, were carried out since these can lead to product loss. Additionally, the DHB matrix used for the MALDI-TOF experiments has a significant peak at 550 Daltons which is close enough in size to many of the oligosaccharides being analyzed to potentially interfere with the detection of the desired oligosaccharide masses. For these reasons, standard reactions were carried out overnight for at least 16 h to enable clear product detection despite the additional presence of contaminating background signals from the buffer and matrix.

### Quantification of Fucosyltransferase Activity Using the GDP-Glo™ Glycosyltransferase Assay Kit

Transferase activity was also measured using the GDP-Glo™ GT Assay Kit (Promega) according to the manufacturer’s instructions. The GDP-Glo™ Kit measures activity based on the amount of GDP produced as a by-product of FUT activity. Standard 5 μl reactions were prepared in 25 mM HEPES, pH 7.2, and consisted of 100 μM GDP-Fuc as the donor, 250 μM of the synthetic AG oligosaccharides (55, 65, 68, 69, and 70; Figure 2A), or the XyG mixture as acceptors (Figure 2C), and 100 ng of enzyme. Assays were initiated with the addition of enzyme and were carried out for a period of 20 min at 24°C. Control assay contained the same components except for the acceptors or the enzyme.

To measure the amount of GDP produced, 5 μl reactions were incubated for an hour at room temperature with equal volumes of GDP-Glo Detection Reagent in a 384-well white, polystyrene, low volume plate (Corning Inc., https://corning.com). Luminescence values were obtained by reading the assay plate with a GloMax® Microplate Reader (Promega). Enzyme activity was quantified using a GDP standard curve according to the manufacturer’s instructions.

### Structural Analysis of FUT Reaction Products by NMR Spectroscopy and Glycosyl Linkage Analysis

NMR experiments were carried out to determine the structure of select fucosylated reaction products. Due to the limited amounts of the AG oligosaccharides (55, 65, 68, 69, and 70) available, NMR experiments were only done with the α-(1,5)-linked arabinan oligosaccharides. Experiments were recorded at 25°C with a Varian Inova NMR spectrometer at 600 MHz using a 5 mm cold probe. Reactions consisted of 50 mM sodium phosphate buffer, pH 7.2, 1 mM of GDP-Fucose, 2 mM of the α-(1,5)-linked arabinan oligosaccharides (Figure 2B), and 500 ng of enzyme. Reactions were left to incubate at 37°C for at least 2 days to reach completion. After lyophilization, 200 μl of D_2_O was added to the samples and were re-lyophilized twice. Samples were dissolved in D_2_O a third and final time and placed in a 3 mm NMR tube. The two-dimensional spectra (COSY and NOESY) were recorded using standard Varian pulse programs. Chemical shifts are given in ppm relative to internal dimethyl sulfoxide (DMSO) standard (*δ*^1^H 2.721). The NMR spectra were processed using MNova software (Mestrelab Research S.L., Santiago de Compostela, Spain). Glycosyl linkage analysis was performed on the reaction products of *At*FUT4 using arabinobiose, arabinotriose, or arabinotetraose as acceptors. For glycosyl linkage analysis, the samples were permethylated, depolymerized, reduced, and acetylated, and the resultant partially methylated alditol acetates (PMAAs) were analyzed by gas chromatography-mass spectrometry (GC-MS). The procedure is a slight modification of the one described by Heiss et al. (2009). Briefly, 1 mg of sample was carefully weighed into borosilicate test tubes with Teflon lined screw caps, suspended in 200 μl of DMSO, and stirred overnight for 16 h. The samples were permethylated using sodium hydroxide (NaOH) and iodomethane (MeI). Following sample workup, the permethylated material was hydrolyzed using 2 M trifluoroacetic acid (2 h in sealed tubes at 121°C), reduced with sodium borodeuteride (NaBD_4_), and acetylated using acetic anhydride/trifluoroacetic acid. The resulting PMAAs were analyzed on an Agilent 7890A GC interfaced to a 5975C mass selective detector (MSD; electron impact ionization mode), and separation was performed on Supelco 2331 fused silica capillary column (30 m × 0.25 mm ID).

### Generation of *AtFUT4*::GUS and *AtFUT6*::GUS Transgenic Plants and GUS Staining

To study the expression pattern and localization of *AtFUT4 in planta*, an *AtFUT4*::GUS fusion-reporter gene was constructed and transformed into WT *A. thaliana* plants for subsequent GUS staining and visualization (Jefferson et al., 1987). To construct the *AtFUT4*::GUS fusion reporter line, primers including restriction sites for *Bam*HI and *Hind*III were used to PCR amplify a ~2,500-base pair fragment upstream of the *AtFUT4* open reading frame using WT (Col-0) *A. thaliana* genomic DNA as a template (Primer sequences listed in Supplementary Table S1). The amplified region, presumably containing the *AtFUT4* native promoter, was then cloned into the pBI101 plant transformation vector in-frame with the GUS reporter gene (Jefferson et al., 1987). The resulting vector was sequenced to ensure the inclusion of the *AtFUT4* promoter and 5' UTR, after which it was used to transform GV3101:PM90 *Agrobacterium tumefaciens* through electroporation (Jefferson et al., 1987). Positively transformed *Agrobacterium* colonies were selected on Luria Broth (LB) plates with 50 μg/ml rifampicin, 25 μg/ml gentamycin, and 50 μg/ml kanamycin, and were verified by colony PCR using a combination of primers that anneal to the *AtFUT4* promoter region as well as to the GUS gene (Supplementary Table S1). Once transformed and verified, the *Agrobacterium* cells containing the *AtFUT4* promoter region were used to transform WT *A. thaliana* using the floral dip method (Clough and Bent, 1998). Successfully transformed *AtFUT4::GUS* plants (*n* > 15) were identified by growing seeds on Murashige and Skoog (MS) media supplemented with 2% (w/v) sucrose and 50 μg/ml kanamycin. *AtFUT6*::GUS transgenic lines had been produced previously in the lab following the same method as was used for the generation of the *AtFUT4::GUS* plants described here. Multiple independent plant lines were generated for *At*FUT4::GUS and *At*FUT6::GUS. The primers used to amplify the promoter regions of both genes can be found in Supplementary Table S1.

GUS transformed seedlings were stained with an X-Gluc staining solution consisting of 50 mM phosphate buffer, pH 7.5, 0.5 mM ferricyanide, 0.5 mM ferrocyanide, 2 mM X-Gluc, 0.05% (v/v) Triton X, and 15% (v/v) methanol (Jefferson et al., 1987). When seedlings had reached the desired age for staining (12 days after sowing), they were submerged in X-Gluc staining solution and placed in a vacuum chamber for a minimum of 30 min, and then incubated at 37°C for 4–6 h to obtain the desired level of staining. The X-Gluc staining solution was then removed, and the seedlings were washed with 70% (v/v) ethanol a minimum of three times to remove any excess stain and chlorophyll. Stained seedlings were imaged with an Olympus dissecting microscope at 40X magnification. Two representative, independently transformed lines are shown for each construct.

## Results

### *At*FUT4 and *At*FUT6 Display Acceptor Substrate Selectivity *in vitro*

The globular catalytic domains of *At*FUT4 and *At*FUT6 were produced and expressed by transiently transfecting mammalian HEK293 suspension-cultured cells utilizing a fusion protein system that has been successfully used for heterologous expression of both mammalian and plant FUTs (Meng et al., 2013; Urbanowicz et al., 2017; Moremen et al., 2018). Expression and secretion of GFP-*At*FUT4 and GFP-*At*FUT6 in HEK293 cells yielded high levels of secreted recombinant fusion protein, based on GFP fluorescence, ~108 and ~77.8 mg L^−1^, respectively (Supplementary Table S1). These expression levels are similar to that observed with GFP-*At*FUT1 in HEK293 cells (120 mg L^−1^; Urbanowicz et al., 2017). Recombinant GFP-*At*FUT4 and GFP-*At*FUT6 fusion proteins were purified by immobilized metal affinity chromatography using Ni^2+^-NTA, resulting in a purity of ≥95% for each protein (Supplementary Figure S1). Both proteins were highly soluble before, during and after purification and buffer exchange.

To determine if the recombinant GFP-*At*FUT4 and GFP-*At*FUT6 fusion proteins maintained the same acceptor substrate specificity as had been determined in a previous study (Wu et al., 2010), five structurally distinct AG-related oligosaccharides (55, 65, 68, 69, and 70), synthesized by automated glycan assembly, were evaluated for their ability to serve as acceptor substrates (Bartetzko et al., 2015; Ruprecht et al., 2017; Bartetzko and Pfrengle, 2019; Figure 2A). Specifically, these acceptors were chosen due to their structural similarities to the previously characterized fucosylated AGP side-chains identified in WT *A. thaliana*: FucAraGal_3_, FucAraGal_4_, and FucXylAraGal_4_ (Tryfona et al., 2012, 2014; Figure 1C). The five AG oligosaccharides selected contain minor differences in structure, such as the presence or absence of a β-(1,6)-linked Gal backbone, the length of the β-(1,3)-linked Gal side-chain, the presence of an α-(1,3)- and α-(1,5)-linked Ara, and/or the terminal or internal positioning of an α-(1,3)-linked Ara on the Gal side-chain (Figure 2A). These structural differences were selected in an attempt to determine whether GFP-*At*FUT4 and GFP-*At*FUT6 would have the same or differing specificity, and to determine if the presence or absence of the Gal backbone, the length of the Gal side-chain, and/or the positioning and linkage of the Ara residue would impart differences on the ability of GFP-*At*FUT4 and GFP-*At*FUT6 to fucosylate these oligosaccharides. The GFP-*At*FUT4 and GFP-*At*FUT6 fusion proteins were also tested against a XyG oligosaccharide mixture consisting of XXXG, XXLG, and XLLG (Pauly et al., 2001; Wu et al., 2010; Tuomivaara et al., 2015; Figure 2C). GFP-*At*FUT1, the XyG-specific member of the GT37 FUT family in *A. thaliana*, was similarly assayed against all AG and XyG oligosaccharides, and was included in this study as a control to probe acceptor substrate specificity of GT37 members.

**Figure 3:**
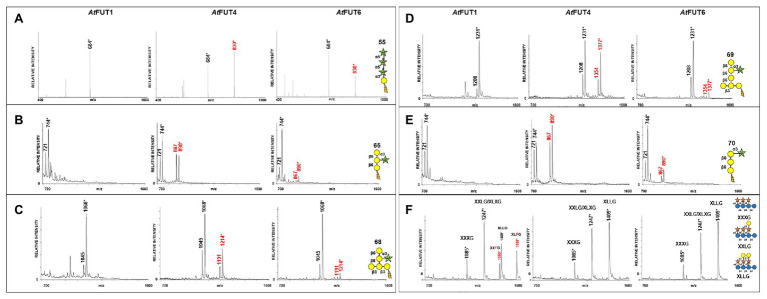
Matrix-assisted laser desorption/ionization-time of flight mass spectrometry (MALDI-TOF MS) data for GFP-*At*FUT1, GFP-*At*FUT4, and GFP-*At*FUT6 reacted with different acceptor substrates. **(A)** Acceptor 55, **(B)** Acceptor 65, **(C)** Acceptor 68, **(D)** Acceptor 69, **(E)** Acceptor 70, and **(F)** XG acceptors. Transfer of Fuc increases the mass of the acceptor by 146 Da, as indicated by annotating (M + H^+^) ions, (^*^) denotes (M + Na^+^) adducts.

Fucosyltransferase activities for GFP-*At*FUT4, GFP-*At*FUT6, and GFP-*At*FUT1 were determined by incubation with the AG and XyG oligosaccharides for a prolonged period, at least 16 h at 24°C, to allow for clear product detection in the presence of background signals arising from the reaction buffer and DHB matrix. The resulting saccharide reaction products, if present, were detected by MALDI-TOF MS. The reaction products were consistent with the transfer of a single Fuc residue onto the selected acceptors based on the observation of the appearance of products with an increased mass of 146 Da corresponding to the addition of a deoxyhexose. Analysis of the reaction products showed that GFP-*At*FUT4 and GFP-*At*FUT6 added a single fucosyl residue to each of the AG oligosaccharides in tested (Figures 3A–E). In contrast, GFP-*At*FUT1 was not active on the AG oligosaccharides, but added Fuc to galactosylated XyG oligosaccharides, as was observed in previous studies (Perrin et al., 1999; Wu et al., 2010; Urbanowicz et al., 2017; Figure 3F).

**Figure 4:**
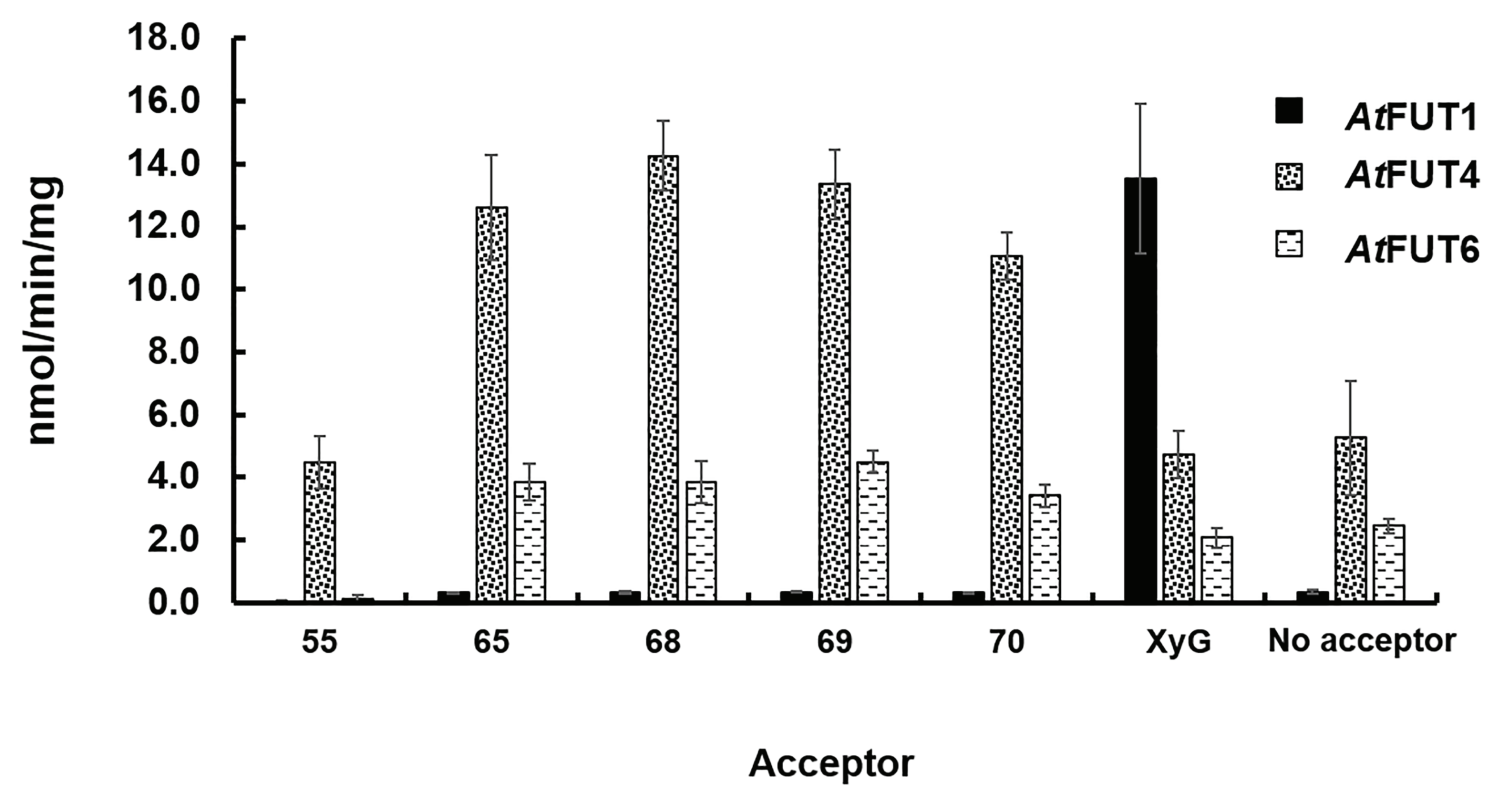
Biochemical analysis of GFP-*At*FUT1, GFP-*At*FUT4, and GFP-*At*FUT6. Enzymatic activity was measured based on the production of GDP using the GDP-Glo assay kit in the presence or absence (Hydrolysis) of acceptor substrates (Figure 2). Values are represented as the average of three technical replicates ±SD, and are reported in nmol/min/mg of protein.

Findings by Wu et al. (2010) suggested that the site of fucosylation for AGPs may lie on an Ara*f* residue. In our study, the only structural commonality between all AG oligosaccharides tested as acceptors was the presence of a terminal Ara*f* residue. The ability of GFP-*At*FUT4 and GFP-*At*FUT6 to fucosylate all the AG oligosaccharides tested, regardless of their structural differences, suggests that GFP-*At*FUT4 and GFP-*At*FUT6 are less selective *in vitro* than previously reported. However, GFP-*At*FUT4 and GFP-*At*FUT6 do appear to maintain their specificity for fucosylating arabinofuranose residues, as they did not fucosylate the galactopyranose residues of XyG, nor did they fucosylate either of the galactan oligosaccharides tested here. GFP-*At*FUT4 had higher apparent activity than GFP-*At*FUT6 (Figure 4) with all acceptors tested, while in previous studies with microsomal *At*FUT4 and *At*FUT6, *At*FUT6 was reported to display more activity than *At*FUT4 (Wu et al., 2010). The differences in protein purification, construct design, and/or assay conditions between the previous study and ours may account for these observed differences in activity. However, we will note that both GFP-*At*FUT4 and GFP-*At*FUT6 were expressed and secreted at high levels and were purified prior to use in the current study (Supplementary Table S1; Supplementary Figure S1).

Glycosyltransferase reactions result in the production of two products: the glycosylated acceptor and the nucleotide from the nucleotide-sugar donor, in this case GDP. The Glo™ system measures transferase activity indirectly, by converting the GDP that is released during transfer into ATP. An enzyme-linked luciferase/luciferin reaction then converts the ATP to luminescence, which can be correlated back to the GDP concentration using a GDP standard curve. Hydrolytic activity can also be measured since, as has been demonstrated before, some GTs can hydrolyze their nucleotide-sugar donor when an acceptor is unavailable. This corresponds to the enzymatic transfer of the sugar from the nucleotide-sugar donor to a water molecule in the absence of an acceptor (Sheikh et al., 2017).

**Figure 5:**
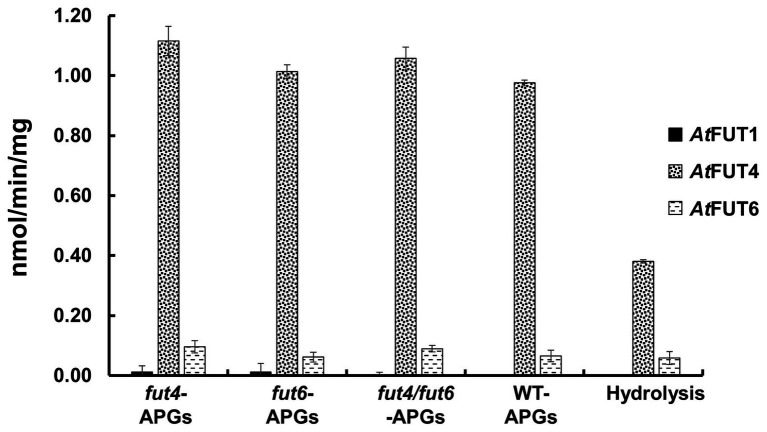
Relative enzyme activity of GFP-*At*FUT1, GFP-*At*FUT4, and GFP-*At*FUT6 using AGPs extracted from vegetative tissue of wild-type (WT), *fut4*, *fut6*, and *fut4fut6 A. thaliana* mutants as acceptor substrates. Values are represented as the average of three technical replicates ±SD, and are reported in nmol/min/mg of protein.

The transferase and hydrolase activities of GFP-*At*FUT4 and GFP-*At*FUT6 were quantified using the GDP-Glo™ kit. The transferase activity data were consistent with the results of the MALDI-TOF MS analysis of the saccharide reaction products. GFP-*At*FUT4 and GFP-*At*FUT6 had detectable activity with the five AG oligosaccharides, but not with the XyG oligosaccharide mixture (Figure 4). Similarly, GFP-*At*FUT1 had detectable activity with the XyG oligosaccharides, but not with the other substrates (Figure 4). As was observed with the MALDI-TOF MS analysis, more transferase activity was detected for GFP-*At*FUT4 than for GFP-*At*FUT6. Previous studies on *At*FUT4 and *At*FUT6 utilized AGPs extracted from their correspondent mutants as acceptor substrates to test the activities of these enzymes (Wu et al., 2010). The AGPs of *fut4* and *fut6* mutants have reduced levels of fucosylated AGPs, and the AGPs of *fut4/fut6* mutants have no detectable fucosylation, as compared to AGPs from WT *A. thaliana* plants (Tryfona et al., 2014). Here, we also extracted AGP-enriched fractions from WT, *fut4*, *fut6*, and *fut4/fut6* single and double mutant plants, and used them as acceptor substrates in the GDP-Glo™ assays (Figure 5). As expected from the data collected in our previous experiments, GFP-*At*FUT1 had no detectable activity with any of the AGP-enriched fractions. In our hands, GFP-*At*FUT4 and GFP-*At*FUT6 showed activity against the AGP fractions, but they did not show any specific preference toward any of the AGP fractions. We cannot exclude the possibility that the AGP extraction method we utilized also extracted other polysaccharides, such as RG-I, that may have Ara-rich side-chains that GFP-*At*FUT4 and GFP-*At*FUT6 can recognize and fucosylate *in vitro*. This untargeted enrichment could possibly explain the lack of specificity detected in this assay.

Among all the assays performed, GFP-*At*FUT4 and GFP-*At*FUT6 exhibited unexpectedly high hydrolytic activity toward GDP-Fuc in the absence of oligosaccharide acceptors (Figures 4, 5). To confirm the unexpectedly high rate of GDP-Fuc hydrolysis observed, hydrolysis assays were also performed with two UDP sugar nucleotides, UDP-xylose and UDP-GalA, using the GDP-Glo™ and UDP-Glo™ assay kits. As compared to GDP-Fuc, a low level of UDP-Xyl hydrolysis was observed with GFP-*At*FUT4. No appreciable hydrolytic activity was observed with either UDP sugar for GFP-*At*FUT6 (Supplementary Figure S2). Unlike GFP-*At*FUT4 and GFP-*At*FUT6, GFP-*At*FUT1 had very low rates of GDP-Fuc hydrolysis, which is consistent with previous findings using the GDP-Glo™ assay system to measure its activity (Urbanowicz et al., 2017). At this time, we do not have an explanation for the high observed rates of GDP-Fuc hydrolysis for GFP-*At*FUT4 and GFP-*At*FUT6, but we hypothesize that the active sites of GFP-*At*FUT4 and GFP-*At*FUT6 may be shallower than the active site of GFP-*At*FUT1, consistent with the ability of these enzymes to use multiple acceptor substrates. A shallower active site would presumably be more accessible to water, facilitating a higher rate of transfer from the nucleotide-sugar donor to a water molecule.

### *At*FUT4 and *At*FUT6 can Fucosylate α-(1,5)- and α-(1,3)-Linked Arabinofuranose Residues

The activities of GFP-*At*FUT4 and GFP-*At*FUT6 were also tested in a high-throughput assay using a micro-array populated with more than 100 synthetic oligosaccharides (Ruprecht et al., 2020). However, the micro-array we used previously can be used to rapidly, identify potential acceptor substrates, but it does not provide any information regarding the structure of the reaction products. Thus, additional analyses are required to determine the final carbohydrate structure of the saccharide reaction products. Based on the AG-like acceptor substrates evaluated herein and in Ruprecht et al. (2020), we observed that the only structural commonality apparently necessary for GFP-*At*FUT4 and GFP-*At*FUT6 to fucosylate the acceptor was the presence of a terminal α-Ara*f* residue in the oligosaccharide. The presence or absence of a β-(1,6)-linked Gal backbone and the length of the β-(1,3) linked Gal side-chain on which the Ara*f* residue is located, did not appear to impart any selectivity. To further investigate acceptor substrate specificity, a series of commercially available Ara- and Gal-containing oligosaccharides were evaluated. Due to the apparent sole selectivity of GFP-*At*FUT4 and GFP-*At*FUT6 for Ara*f*, three commercialy available α-(1,5)-linked arabinan oligosaccharides, including arabinobiose, arabinotriose, and arabinotetraose, were selected as possible acceptor substrates (Figure 2B). The three α-(1,5)-linked arabinan oligosaccharides are of varying lengths and were chosen to determine the minimum length that GFP-*At*FUT4 and/or GFP-*At*FUT6 are able to utilize. Furthermore, two galacto-oligosaccharides, β-(1,3) galactobiose and β-(1,6) galactobiose, were also used to conclusively eliminate galactose residues as the site of fucosylation (Figure 2D). No α-(1,3)-linked arabino-oligosaccharides were commercially available at the time of this study.

**Figure 6:**
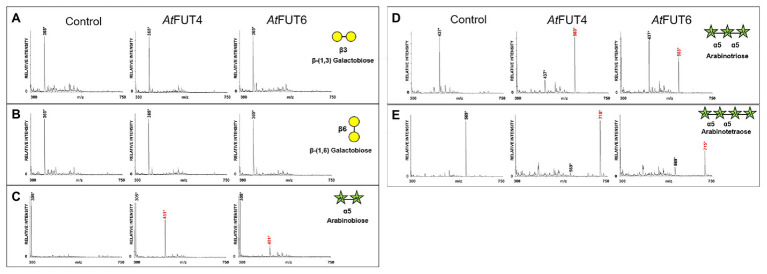
MALDI-TOF MS analysis of products generated by incubating GFP-*At*FUT4, and GFP-*At*FUT6 with GDP-Fuc and different linear arabinan or galactan acceptor substrates. **(A)** β-(1,3)-linked galactobiose, **(B)** β-(1,6)-linked galactobiose, **(C)** α-(1,5)-linked arabinobiose, **(D)** α-(1,5)-linked, arabinotriose, and **(E)** α-(1,5)-linked arabinotetraose. Transfer of Fuc increases the mass of the acceptor by 146 Da, as indicated by annotating (M + H^+^) ions, (^*^) denotes (M + Na^+^) adducts. Control assays contained the same components except for enzyme.

Interestingly, all three α-(1,5)-linked arabinan oligosaccharides were fucosylated by both GFP-*At*FUT4 and GFP-*At*FUT6. MALTI-TOF MS analyses of the reaction products indicated that both GFP-*At*FUT4 and GFP-*At*FUT6 catalyze the transfer of a single Fuc residue to each of the arabinan oligosaccharides based on the observation of structures with a mass increase of 146 Da (Figures 6C–E). In contrast, analysis of the reactions containing β-(1,3) galactobiose or β-(1,6) galactobiose showed no difference relative to control samples lacking enzyme (Figures 6A,B). Taken together, these data indicate that Ara, but not Gal, is the acceptor site of fucosylation for GFP-*At*FUT4 and GFP-*At*FUT6. Furthermore, these results suggest that the galactose residues in the AG oligosaccharides may not be critical components for substrate specificity of these enzymes.

**Figure 7:**
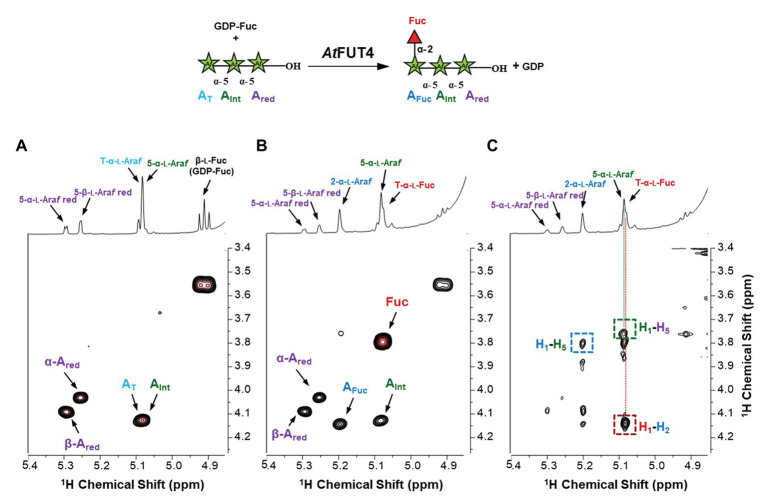
NMR analysis of the products formed when arabinotriose was incubated with GFP-*At*FUT4 and GDP-Fuc. The scheme of the reaction is shown at the top of the figure. The labeled cross-peaks in the two-dimensional COSY spectrum of the control **(A)** correspond to the anomeric signals of the residues of the arabinotriose. After the reaction with GFP-*At*FUT4, the COSY spectrum **(B)** contained two additional signals, which were identified as terminal Fuc and an Ara with Fuc attached at *O*2. The signals surrounded by squares in the NOESY spectrum of the GFP-*At*FUT4 reaction **(C)** indicate the glycosidic linkages in the enzymatically-generated fucosylated oligosaccharide. For the complete list of assignments, see Supplementary Table S2.

**Figure 8:**
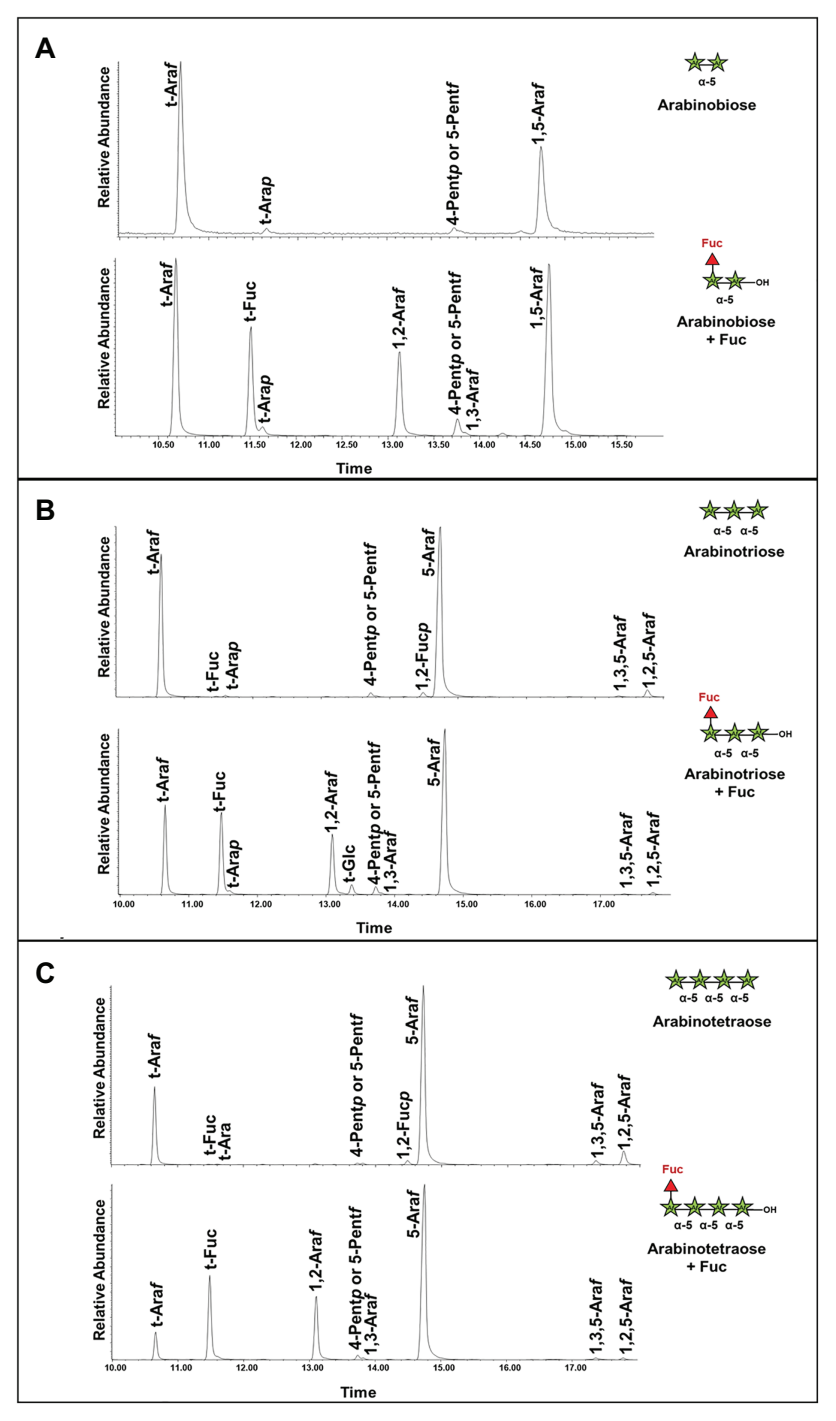
Linkage analysis of the reaction products of arabinan oligosaccharides incubated with GFP-*At*FUT4. **(A)** Unreacted arabinobiose and arabinobiose incubated with GFP-*At*FUT4. **(B)** Unreacted arabinotriose and arabinotriose incubated with GFP-*At*FUT4. **(C)** Unreacted arabinotetraose and arabinotetraose incubated with GFP-*At*FUT4. The presence of the 1,2-Ara*f* peak in the spectra of the reaction products indicates that Fuc is attached to the terminal non-reducing Ara in all the oligosaccharides. As the 1,3,5-Ara*f* and the 1,2,5-Ara*f* peaks appear in the unreacted and reacted spectra we do not ascribe these to transfer in those linkages.

To confirm the structure of the fucosylated arabinans obtained after incubation with the enzymes, NMR analyses were performed (Figure 7). GFP-*At*FUT4 was chosen for this analysis since it has appreciably more activity than GFP-*At*FUT6, and both enzymes showed similar specificity toward the arabinan oligosaccharides. NMR analyses were carried out on the reaction product of GFP-*At*FUT4 with the arabinotriose acceptor substrate, which contains two terminals and only one internal Ara*f* residue. The NMR spectrum of arabinotriose incubated with GFP-*At*FUT4 clearly contained two additional signals that were not present in the spectrum of unreacted arabinotriose (Figures 7A,B). These signals were assigned as terminal Fuc, and Ara with Fuc attached at *O*2 based on the chemical shifts and the cross-peaks in the NOESY spectrum, which indicate the linkages between the residues in the oligosaccharide (Figure 7C; Supplementary Table S2). These results conclusively demonstrate that *At*FUT4 is an α-(1,2) FUT, as is *At*FUT1, and that it catalyzes the transfer of a single Fuc onto terminal Ara residues. To further confirm that Fuc was added only to the terminal Ara for all the arabinan oligosaccharides, glycosyl linkage analyses were performed on the reaction products of *At*FUT4 incubated with arabinobiose, arabinotriose, and arabinotetraose. The presence of peaks for (1,2)-Ara*f* and the absence of (1,2,3)-Ara*f* and (1,2,5)-Ara*f* peaks in all the spectra confirmed the findings from the NMR analyses, and further proved that for arabinobiose and arabinotetraose, Fuc is also added onto the terminal Ara at the non-reducing end of the oligosaccharides (Figure 8).

### GUS Promoter Studies Reveal *At*FUT4 and *At*FUT6 are Differentially Expressed in *Arabidopsis thaliana* Roots

The expression patterns of *AtFUT4* and *AtFUT6* have been previously reported at the whole organ level, with *AtFUT4* being shown to localize to both the leaf and root, and *AtFUT6* localizing only to the root (Sarria et al., 2001). It was accordingly demonstrated that *AtFUT4* is solely responsible for the fucosylation of leaf AGPs, while both *AtFUT4* and *AtFUT6* function in the root to produce fucosylated AGPs (Liang et al., 2013; Tryfona et al., 2014). As both *AtFUT4* and *AtFUT6* are expressed and functional in the root, and produce the same fucosylated structures, we predicted that their co-expression in this tissue may be due to their gene expression and/or gene products sub-localizing to different cell types. Precedents for our prediction have been reported for the previously mentioned *AGM1* and *AGM2* (Temple et al., 2019; Smith et al., 2020), as well as for the two isoforms of GDP-d-mannose 4,6-dehydratase (*GMD1* and *GMD2*) in *A. thaliana* (Bonin et al., 1997, 2003; Bonin and Reiter, 2000).

**Figure 9:**
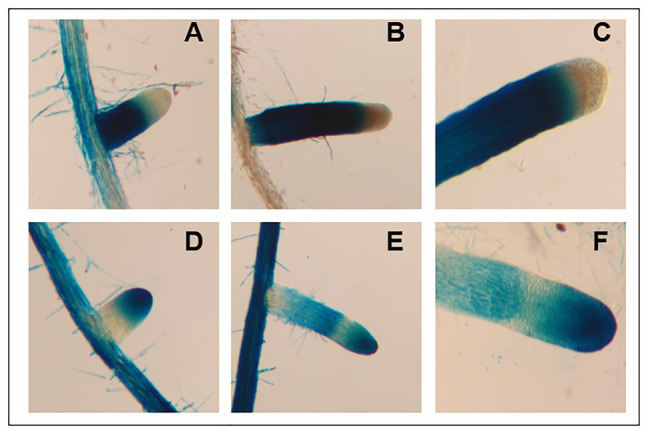
Imaging and staining of 12-day-old *AtFUT4::GUS* and *AtFUT6::GUS* seedlings. Multiple (*n* > 15) independent *AtFUT4::GUS* and *AtFUT6::GUS* plant lines are generated, grown, and observed for each construct. Visualization and qualitative analysis of GUS staining patterns of roots for two representative lines are shown. Staining patterns for lateral roots of increasing lengths are shown in **(A,B,D,E)**, while close-ups of tap roots are shown in **(C,F)**. **(A,B)** *AtFUT4::GUS-1*, **(C)** *AtFUT4::GUS-2*, **(D,E)** *AtFUT6::GUS-1*, and **(F)** *AtFUT6::GUS-2*.

To investigate the cellular gene expression patterns of *AtFUT4* and *AtFUT6*, transgenic plants containing the β-glucuronidase (GUS) reporter gene under control of the native promoter and 5' UTR (~2,500 bp upstream of the start codon) of *AtFUT4* and *AtFUT6* were generated. As expected, *AtFUT4::GUS* and *AtFUT6::GUS* exhibited differing, yet complementary, localization patterns. The first visible difference upon staining was at the tap root and lateral roots of the seedlings, with the root of *AtFUT4::GUS* seedlings staining everywhere except for the elongation and meristematic zones (Figures 9A–C). The tap root of *AtFUT6::GUS* seedlings, on the other hand, stained most visibly in the elongation and meristematic zones (Figures 9D–F). This pattern was repeated in newly formed and emerging lateral roots. The lateral roots of *AtFUT4::GUS* seedlings show strong GUS staining at the base of the lateral root but not at the tip, while the *AtFUT6::GUS* seedlings demonstrate the opposite pattern, exhibiting strong GUS staining only at the tip of the lateral root. The complementary GUS activity patterns detected for *AtFUT4::GUS* and *AtFUT6::GUS* suggest that *AtFUT4* and *AtFUT6* are cell-type specific in the root, and thus have differing physiological roles *in planta*.

## Discussion

Multiple lines of evidence are presented here to provide an updated view on the activities and substrate specificities of *At*FUT1, *At*FUT4, and *At*FUT6. In contrast to previous findings, our data show that *At*FUT4 and *At*FUT6 have the same donor and acceptor substrate specificity and identically recognize various AG-like and non-AG-like oligosaccharides and polysaccharides as acceptor substrates (Figures 3, 5, 6). One critical trend that we observed is that *At*FUT4 and *At*FUT6 recognize both α-(1,3) and α-(1,5) linked Ara residues on AG-like and non-AG-like oligosaccharides and polysaccharides (Ruprecht et al., 2020), and do not fucosylate the β-(1,6)-linked Gal backbone or the β-(1,3)-linked Gal side-chains of their native targets, AGPs (Figure 6).

As demonstrated in this study, GFP-*At*FUT4 and GFP-*At*FUT6 appear to have much broader and less stringent specificities than GFP-*At*FUT1. *At*FUT1 has been demonstrated to fucosylate only the β-(1,2)-linked Gal residues of tamarind (Perrin et al., 1999) and *A. thaliana* XyG (Urbanowicz et al., 2017), and the β-(1,2)-linked galacturonic acid (GalA) residues of root hair XyGs on *O*2 of the Xyl residues closest to the reducing end of the XyG oligosaccharide (Peña et al., 2012; Figure 1C). In our *in vitro* studies and in the glycoarray study by Ruprecht et al. (2020), GFP-*At*FUT4 and GFP-*At*FUT6 recognize and fucosylate Ara in both α-(1,3) and α-(1,5) linkages, so long as those Ara residues are not on internal Gal residues of a 3-linked β-Gal oligosaccharide. Otherwise, GFP-*At*FUT4 and GFP-*At*FUT6 can add fucose to free Ara*f* independently of the structure of the saccharide they are appended to. Additionally, GFP-*At*FUT4 and GFP-*At*FUT6 were shown to fucosylate an identical subset of the oligo- and polysaccharides in the glycoarray assay, further suggesting that they have identical specificity (Ruprecht et al., 2020). Both enzymes show selectivity for arabinogalactan structures; however, none of the structurally-related galactan structures nor other unrelated structures tested are fucosylated by these enzymes (Figure 2D; Ruprecht et al., 2020).

We recognize that reaction lengths of 16 h are unusual, and out of most physiological ranges, but these reactions were performed to identify if product formation could occur with the various oligosaccharides we selected for this study. Furthermore, we recognize that performing enzyme kinetics assays would provide more accurate representations of the activities of *At*FUT4 and *At*FUT6 *in vivo*. Due to the limited amounts of the oligosaccharides available for this study, and our downstream interests in utilizing these enzymes for the creation of oligo- and polysaccharides with specific modifications, we felt detailed enzyme kinetics to be out the scope of the current study. Regardless, we can conclusively report that while GFP-*At*FUT4 and GFP-*At*FUT6 recognized the same oligosaccharides as acceptors in our various *in vitro* assays, as well as in the more comprehensive glycoarray assay (Ruprecht et al., 2020), GFP-*At*FUT4 was consistently more active than GFP-*At*FUT6 (Figures 3–6). The arabinogalactan oligosaccharides that we have tested and that were included in the glycoarray studies (Ruprecht et al., 2020) are based on the limited structural information about arabinogalactan glycans currently available (Kieliszewski, 2001; Tryfona et al., 2012, 2014). Thus, we cannot dismiss the possibility that *At*FUT6 may have preferences for an arabinogalactan substrate we have not considered. *At*FUT6 may also require an additional cofactor(s) that has yet to be identified, and that is not required by *At*FUT4. We also cannot omit the possibility that *At*FUT6 may be fucosylating a yet unidentified Ara residue from another cell wall glycan, such as RG-I, which also has prominent AG side-chains. Finally, we presume, but have not validated, that GFP-*At*FUT6 is fucosylating the various oligosaccharides tested, in an α-(1,2)-linkage as has now been shown for GFP-*At*FUT4. Future experiments to optimize the activity of GFP-*At*FUT6 may serve to characterize *At*FUT6 and its intricacies in even more detail.

Despite the broader scope of acceptor substrates that GFP-*At*FUT4 and GFP-*At*FUT6 recognize, our results showed that GFP-*At*FUT4, and most likely also GFP-*At*FUT6, fucosylate the acceptor forming an α-(1,2)-linkage (Figure 8). Furthermore, we show that the minimum requirement for fucosylation by GFP-*At*FUT4 and GFP-*At*FUT6 appears to be the disaccharide arabinobiose (Figure 6), while that of GFP-*At*FUT1 is much larger. Significant differences in the active sites of *At*FUT4 and *At*FUT6, as compared to the active site of *At*FUT1, may account for the broader diversity of reactions that *At*FUT4 and *At*FUT6 can catalyze. For example, the active sites of *At*FUT4 and *At*FUT6 may be shallower than that of *At*FUT1, allowing them to accommodate a wider array of oligosaccharides and polysaccharides. In contrast to *At*FUT1, *At*FUT4 and *At*FUT6 may use an amino acid residue in the catalytic cleft as a catalytic base, which may explain the higher observed propensity of GFP-*At*FUT4 and GFP-*At*FUT6 to hydrolyze GDP-Fuc than detected for GFP-*At*FUT1 (Figure 4). Structural biology efforts are underway to investigate these enzymes in more detail.

Our results, taken together, are not consistent with previous findings suggesting that *At*FUT4 and *At*FUT6 are non- or only partially-redundant, and rather suggest that *At*FUT4 and *At*FUT6 recognize the same structures as suitable acceptors *in vitro*, as both fucosylate the same oligosaccharides among those tested. Despite the apparent redundancies in the specificities of the *At*FUT4 and *At*FUT6 proteins *in vitro*, promoter-reporter gene studies show that *AtFUT4* and *AtFUT6* gene expression patterns sub-localize to different areas of the root *in planta* (Figure 9). As with other proteins that have been demonstrated to have cell-type specific expression patterns, for example *GMD1* and *GMD2* (Bonin et al., 2003), *AtFUT4* and *AtFUT6* are expressed in two distinct cellular regions of the plant root (Figure 9). As *AtFUT6* is only expressed in the root tip (Figures 9D,E), this would suggest that *AtFUT4* is the major contributor to AGP fucosylation in this organ, while *AtFUT6* is functional in only a subset of cell types at the root tip. Studies on *fut4/fut6* double mutants, however, point to the requirement of both genes for proper AGP fucosylation in order to maintain cell expansion under salt-stress conditions (Tryfona et al., 2014).

The findings from this study provide an important update on the function and specificities of two GT37 FUTs, *At*FUT4 and *At*FUT6. Additionally, another GT37 FUT, *At*FUT7, has recently been shown to also be an active FUT with similar, though less extensive, selectivity for arabinogalactan oligosaccharides to *At*FUT4 and *At*FUT6 (Ruprecht et al., 2020). Detailed studies on the specificities of *At*FUT7 may serve to decipher some of the discrepancies we observed between the activity levels of *At*FUT4 and *At*FUT6. It may be possible that *At*FUT4 plays a major role in AGP fucosylation, while *At*FUT6 and *At*FUT7 have more specialized, cell-type specific roles in the fucosylation of AGPs and/or other fucosylated cell wall structures. Regardless, the combination of a broad glycan microarray assay followed with more detailed biochemical assays is a powerful technique for the discovery of novel plant GT functions. Applying this technique to the remaining members of the GT37 family may aid in the identification of the as-of-yet undetermined FUTs specific for the fucosylation of RG-I and RG-II. Altogether these discoveries serve to further our understanding of the plant-specific GT37 family, and provide further proof that previous struggles to study and functionally characterize plant GTs can be overcome. Finally, the broader specificity of functions identified for *At*FUT4 and *At*FUT6 will aid in the creation of specific modifications of plant cell wall structures and other, non-plant derived glycans.

## Data Availability Statement

The raw data supporting the conclusions of this article will be made available by the authors, without undue reservation, to any qualified researcher.

## Author Contributions

MS designed and performed FUT activity assays and promoter-GUS studies. PP and H-TW purified proteins and designed and performed FUT activity assays. DC performed HEK293 transfections. JB grew Arabidopsis lines and performed cell wall fractionation. MB chemically synthesized AG saccharide acceptors. IB and PA performed linkage analysis. MP carried out NMR analysis and interpreted data. FP and KM designed experiments and wrote the manuscript. MS, BU, and MH conceived the study and wrote the manuscript. All authors contributed to the article and approved the submitted version.

### Conflict of Interest

The authors declare that the research was conducted in the absence of any commercial or financial relationships that could be construed as a potential conflict of interest.
