# Supplementary material for: AtFUT4 and AtFUT6 Are Arabinofuranose-Specific Fucosyltransferases
Source: Front Plant Sci. 2021 Feb 9;12:589518. doi: 10.3389/fpls.2021.589518 (PMC7900004; doi:10.3389/fpls.2021.589518)
Supplement: Supplementary file 1 [file Data_Sheet_1.pdf]

# *Supplementary Material*

# *At*FUT4 and *At*FUT6 are Arabinofuranose-specific Fucosyltransferases

**Maria J. Soto[1,2,3], Pradeep Kumar Prabahkar[2,4,5], Hsin-Tzu Wang[2,4,5], Jason Backe[2,4,], Digantkumar Chapla[2,5], Max Bartetzko[6], Ian Black[2], Parastoo Azadi[2,5], Maria J. Pena[2,4], Fabian Pfrengle[6,7], Kelley W. Moremen[2,5], Breeanna R. Urbanowicz[2,4,5*], and Michael G. Hahn[2,3,4*]**

**Supplementary Table 1. Sequences of primers used to amplify truncated GFP-*At*FUT4 and GFP-*At*FUT6, and for the amplification of the native *AtFUT4* and *AtFUT6* promoter regions for subsequent GUS transformation.**

The underlined regions in the primers sequences for AtFUT4 and AtFUT6 specify the partial attB adapter sequences used in the first round of PCR amplification. Expression and secretion of the GFP-AtFUT4 and GFP-AtFUT6 fusion-proteins was determined by measuring the fluorescence of the recombinant proteins in the media used to transiently transfect the HEK293 cells. The concentration of secreted fusion protein in mg/L is shown in parentheses and was estimated based on GFP fluorescence (13.1 flourescence units = 1 mg/L).

| **Enzyme/Gene** | **Amino Acid Truncation** | **Primer Sequence 5' - 3'** | **GFP Fluorescence** |
|---|---|---|---|
| *At*FUT4_F | Δ_54 | AACTTGTACTTTCAAGGCAACGACGAATCCGAAACA | 1423 |
| *At*FUT4_R | | ACAAGAAAGCTGGGTCCTATAACTCATCAAAAAGCT | (108.62 mg/L) |
| *At*FUT6_F | Δ_43 | AACTTGTACTTTCAAGGCAACGACTTCAACAACCAAC | 1028 |
| *At*FUT6_R | | ACAAGAAAGCTGGGTCCTATAACTCATCAAATAGCTTA | (77.8 mg/L) |
| *AtFUT4*::GUS_F | | AAGCTTTTGTGCTCGCTTGAATCAGAAG | |
| *AtFUT4*::GUS_R | | GGATCCGTTGACTTTTAGTTTGTGAAGATGATT | N/A |
| *AtFUT6*::GUS_F | | GGATCCCTTCAAACCAAAAGCTCTG | |
| *AtFUT6*::GUS_R | | AAGCTTATTTTACAAATCGAAACAG | N/A |

**Supplementary Table 2. $^{1}$H NMR signal assignments of arabinotriose incubated with GDP-Fuc and GFP-*At*FUT4.**

(A.) Reaction without the enzyme (control) and after (B) incubation with the enzyme. [1]Chemical shifts are reported in ppm relative to internal dimethyl sulfoxide, $\delta_H$ 2.721.

| **Residue** | **H1** | **H2** | **H3** | **H4** | **H5$_{a,b}$** | **H6** |
|---|---|---|---|---|---|---|
| **Chemical Shift (ppm)[1]** | | | | | | |
| **A.** | | **α-L-Ara*f*-(1,5)-α-L-Ara*f*-(1,5)-α-L-Ara*f*** | | | | |
| T-α-l-Ara*f* | 5.090 | 4.13 | 3.95 | 4.09 | 3.83-3.72 | |
| 5-α-l-Ara*f* | 5.084 | 4.13 | 4.22 | 3.89 | 3.80<br>7.7 | |
| 5-α-l-Ara*f* | 5.258 | 4.03 | 4.03 | 4.23 | 3.86-3.76 | |
| 5-β-l-Ara*f* | 5.294 | 4.09 | 4.00 | 4.23 | 3.86-3.76 | |
| **B.** | | **α-L-Fuc*p*-(1,2)-α-L-Ara*f*-(1,5)-α-L-Ara*f*-(1,5)-α-L-Ara*f*** | | | | |
| T-α-l-Fuc*p* | 5.076 | 3.79 | 3.89 | 3.8 | 4.08 | 1.24 |
| 2-α-l-Ara*f* | 5.196 | 4.14 | 4.10 | 4.10 | 3.835-3.724 | |
| 5-α-l-Ara*f* | 5.084 | 4.13 | 4.22 | 3.89 | 3.80<br>7.7 | |
| 5-α-l-Ara*f* | 5.258 | 4.03 | 4.03 | 4.23 | 3.86-3.76 | |
| 5-β-l-Ara*f* | 5.294 | 4.09 | 4.00 | 4.23 | 3.86-3.76 | |

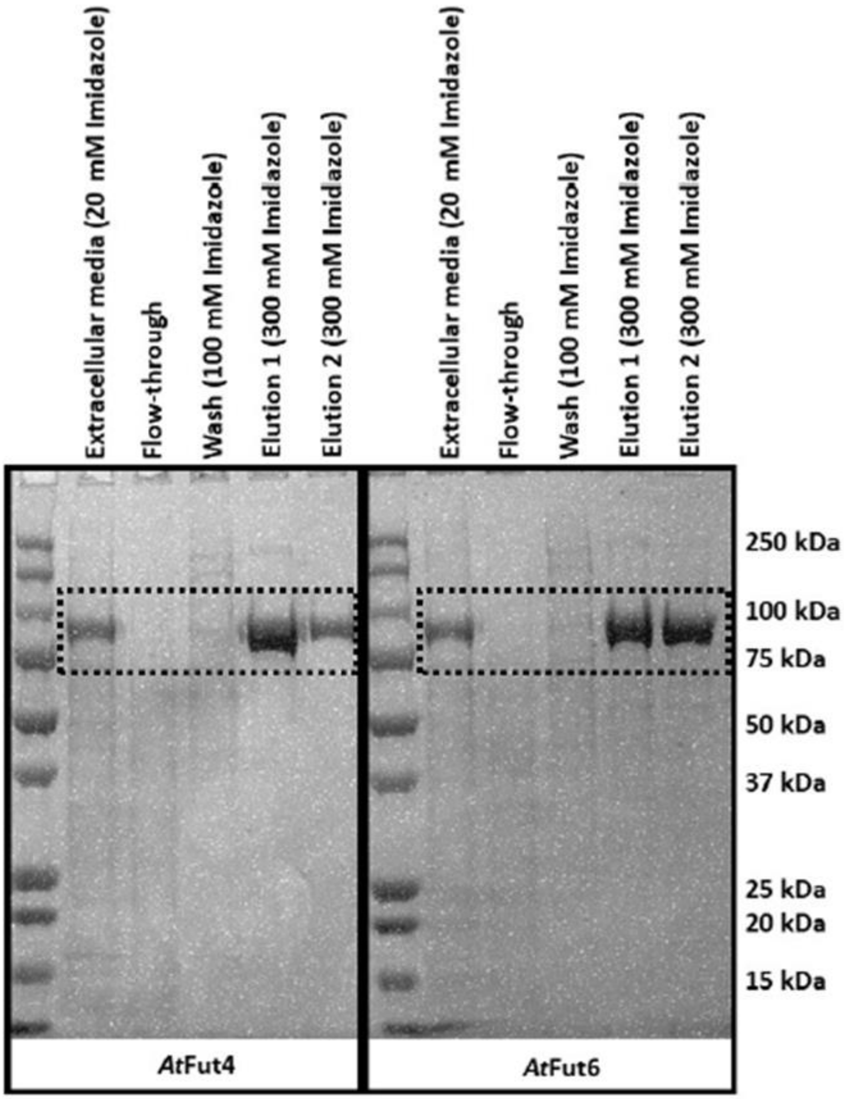


**Supplementary Figure S1. Purification of GFP-*At*FUT4 and GFP-*At*FUT6 using immobilized metal affinity chromatography (IMAC).**

SDS-PAGE of purification of GFP-AtFUT4 and GFP-AtFUT6. Respective protein bands are highlighted in the dashed boxes. All buffers contain 50 mM HEPES and 400 mM NaCl, pH 7.2, and the concentration of imidazole used during the purification is indicated.

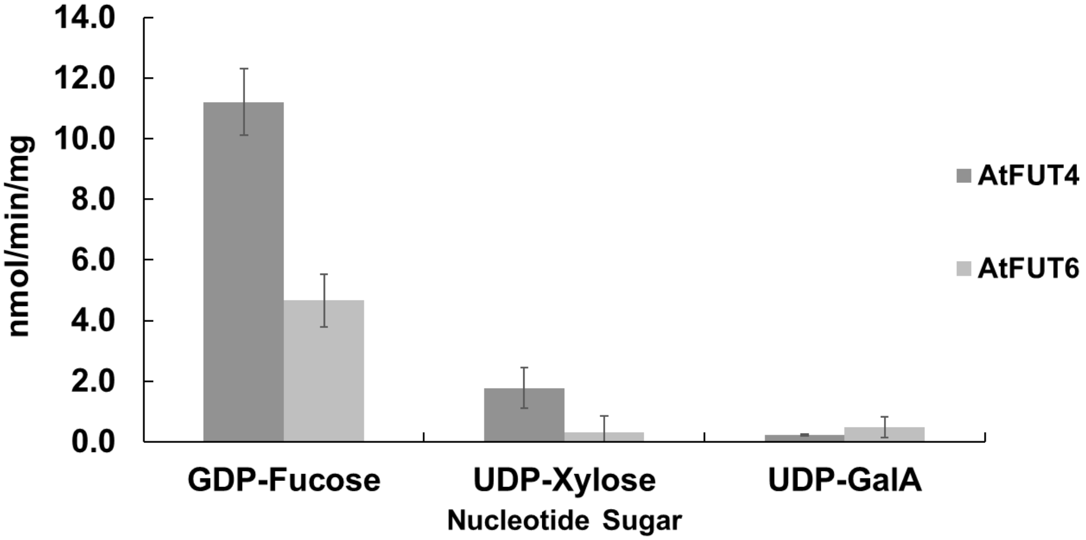


**Supplementary Figure 2. Hydrolysis of GDP-Fucose, UDP-Xylose, and UDP-GalA by GFP-*At*FUT4 and GFP-*At*FUT6.**

Enzyme hydrolysis activity for *At*FUT4, and *At*FUT6 was measured based on the production of GDP or UDP using the GDP-Glo and UDP-Glo assay kits in the absence of acceptor substrates. Enzyme assays consisted of 150 ng of enzyme and 100 µM of nucleotide sugars and were incubated for 20 minutes.
